# NMR Chemical Shift Ranges of Urine Metabolites in Various Organic Solvents

**DOI:** 10.3390/metabo6030027

**Published:** 2016-09-02

**Authors:** Benjamin Görling, Stefan Bräse, Burkhard Luy

**Affiliations:** 1 Institute of Organic Chemistry, Karlsruhe Institute of Technology, Fritz-Haber-Weg 6, Karlsruhe 76131, Germany; benjamin.goerling@kit.edu (B.G.); stefan.braese@kit.edu (S.B.); 2 Institute for Biological Interfaces 4—Magnetic Resonance, Karlsruhe Institute of Technology, Hermann-von-Helmholtz-Platz 1, Eggenstein-Leopoldshafen 76344, Germany; 3 Institute for Toxicology and Genetics, Karlsruhe Institute of Technology, Hermann-von-Helmholtz-Platz 1, Eggenstein-Leopoldshafen 76344, Germany

**Keywords:** NMR spectroscopy, urine, solvents, methanol, DMSO

## Abstract

Signal stability is essential for reliable multivariate data analysis. Urine samples show strong variance in signal positions due to inter patient differences. Here we study the exchange of the solvent of a defined urine matrix and how it affects signal and integral stability of the urinary metabolites by NMR spectroscopy. The exchange solvents were methanol, acetonitrile, dimethyl sulfoxide, chloroform, acetone, dichloromethane, and dimethyl formamide. Some of these solvents showed promising results with a single batch of urine. To evaluate further differences between urine samples, various acid, base, and salt solutions were added in a defined way mimicking to some extent inter human differences. Corresponding chemical shift changes were monitored.

## 1. Introduction

Metabolomics is a powerful tool to analyze differences and perturbations in the metabolic profiles of biological samples like blood plasma/serum, urine, and extracts of tissue, feces, and cells. Especially, nuclear magnetic resonance (NMR) spectroscopy has proven to be an easy and versatile tool to analyze these biological samples [1,2,3]. Urine itself is a highly variable bioliquid with a plethora of endogenous metabolites [4] and very large variations in their signal positions even after the addition of a considerable amount of buffer [5,6]. The variations are mainly subjected to inter-human differences in nutrition and physical condition [6]. Nevertheless, the urine profile of a single person can show strong variations during the day as well. These differences result in altered metabolite concentrations, pH, ionic strength, and amount of divalent ions [7,8,9,10,11]. Values of pH can vary in a range from 4.6 to 8.0 [12] and ionic strength by a factor of 10 [13]. Additionally, in special cases degradation of organic metabolites may occur due to bacterial contamination [14]. Variability in signal position will complicate multivariate data analysis. To reduce the variability in signal position, several approaches have been developed including dilution, adjusting the mixing ratio of urine and buffer and treatment with EDTA (=Ethylenediaminetetraacetic acid) or KF to reduce the effect of the divalent ions Ca^2+^ and Mg^2+^ [9,11,15]. For tissue samples or samples that are extracted prior to analysis, much narrower ranges have been reported [16,17,18,19]. The search for an ideal urine matrix is therefore steadily continuing.

As various organic solvents like methanol and acetonitrile have shown very narrow distributions of metabolite chemical shift ranges in other bioliquids and liquid foods (Bruker, private communication), the idea of solvent exchange to test this hypothesis was born. The aim of this study was therefore to test if urine in different solvents shows less variability in terms of signal positions and increased sample stability in terms of data interpretation.

## 2. Results and Discussion

**Figure 1:**
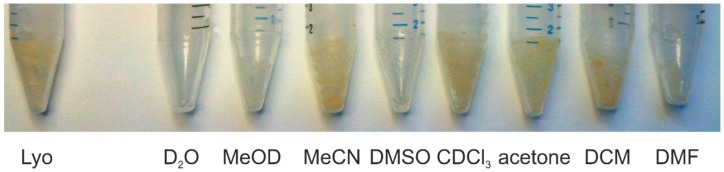
Residue of lyophilized urine samples before (Lyo) and after dissolution in various solvents. With D_2_O as the solvent, no residual remainders are visible, with MeOD, DMSO, and DMF a minor debris is visible and with MeCN, CDCl_3_, acetone, and DCM nearly no difference to the undissolved sample is visible, indicating substantially incomplete dissolution.

To test the reproducibility and the stability of signal positions, urine samples were lyophilized for two days and the residue was dissolved in the desired solvent. The used solvents were D_2_O, methanol-*d*_4_ (MeOD), acetonitrile-*d*_3_ (MeCN), dimethyl sulfoxide-*d*_6_ (DMSO), CDCl_3_, acetone-*d*_6_, dichloromethane-*d*_2_ (DCM), and *N*,*N*-dimethyl formamide-*d*_7_ (DMF). Five individual replicates of a single batch of human urine were prepared for each solvent. Complete dissolution could only be achieved with water as solvent. In the polar solvents MeOD, DMSO, and DMF most of the substances could be dissolved and there was only a minor residue in the centrifuge tube. In the remaining solvents MeCN, CDCl_3_, acetone, and DCM nearly no difference to the freshly lyophilized residue could be observed, indicating insufficient solubility of the urine matrix in these solvents (Figure 1).

Main components in the residue of the polar solvents MeOD, DMSO, and DMF are most likely salts that are only completely soluble in water. Most of the relevant metabolites can be considered to be dissolved and only slight differences in the concentrations should be visible. It should be noted that even substances with low solubility in organic solvents, like for example alanine [20], are still readily dissolved as the concentration present in urine is sufficiently low [21]. As a test, integral values of five exemplarily chosen metabolites were calculated for each solvent. The evaluated metabolites were hippurate, creatinine, lactate, histidine, and alanine. The only metabolite present in all solvents was hippurate, while creatinine and lactate were found in all solvents but chloroform and dichloromethane. Histidine and alanine were solely found in water, methanol, DMSO, and DMF.

**Figure 2:**
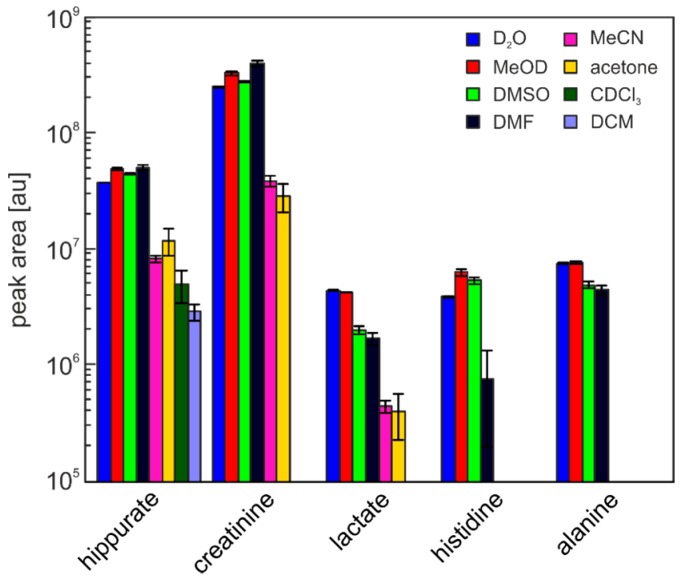
Mean integrals with standard deviations for the selected metabolites.

The relative standard deviation of the integral values shows very good reproducibility for water with values below 2% for all metabolites. Methanol and DMSO show a good reproducibility with relative standard deviations below 7% for methanol and around 10% for DMSO for all metabolites. The last solvent which showed good solubility of the urine matrix, DMF, has a good reproducibility for most of the examined metabolites (relative standard deviation below 10%) but very strong variations in the histidine signal (75%). This is caused by an intensive signal next to the histidine signal that overlaps quite strongly in some samples. Acetonitrile and acetone with at least three of the five evaluated metabolites present, show both strong variations with relative standard deviations of up to 44% for MeCN and 27% for acetone. Although the variations in chloroform and dichloromethane are relatively small, only one of the metabolite was found, eliminating them as suitable solvents in metabolomics-type studies. Results are summarized in Table 1 and Figure 2.

Comparing the intensity of the integrals to the values in water (Table 2), it is striking that some integrals are higher in MeOD, DMSO, and DMF as compared to the original urine sample. It can be assumed that all metabolites are completely dissolved in water after freeze drying and we cannot think of any reason why this should not be the case here. Slight differences in the integral values can occur due to strongly varying baseline levels for all solvents and an imperfect baseline subtraction prior to integration. Given the strong differences for example in methanol (1.3 for hippurate and creatinine and 1.6 for histidine), there must be an effect of the solvents. The most probable explanation is a reduction of the T_1_ time of the metabolites, resulting in a better polarization recovery into the equilibrium state and thereby more intense signals when using the identical duration of the experiment. Indeed, measurement of T_1_ times on a separate urine sample lyophylized and redissolved in the various solvents indicates relaxation times larger than 2 s for almost all metabolites investigated in D_2_O, while they are shorter in MeOD, DMSO, and DMF (Figure S1). Considering the applied recovery delay of 4 s and acquisition time of approximately 2.6 s as commonly applied periods in urine NMR studies, insufficient relaxation especially of histidine, hippurate, and creatinine are expected.

**Figure 3:**
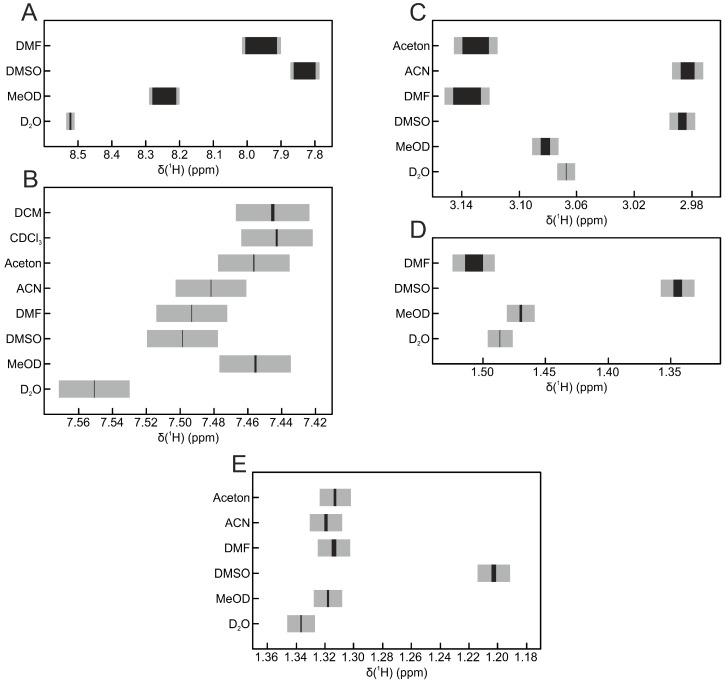
Signal positions of selected metabolites. The black bar represents the center of the signal and its shift over all replicates. The grey box represents the area of the whole signal over all replicates. (**A**) histidine; (**B**) hippurate; (**C**) creatinine; (**D**) alanine; (**E**) lactate.

The variation in signal position for the five replicates for each solvent is shown in Figure 3. The signal positions of the metabolites lactate and hippurate seem to be very robust, because there are only minor shifts in the signal position visible. Still, shifts of more than 0.1 ppm can be observed between the different solvents. The same trend can be seen for alanine and creatinine but with stronger variations in the signal position within individual solvents. As expected, the biggest variations can be observed in the histidine signal: between solvents, its position shifts over a range of more than 0.7 ppm, while within one solvent, the variation can be up to 0.1 ppm (DMF). While differences in the chemical shift values between the solvents are expected and known [22], variation within one solvent should be considered to be low. Interestingly, in all cases, the smallest variations of signal positions were found in water.

Given the bad solubility of the urine matrix in chloroform and dichloromethane, the big relative standard deviation in acetone and acetonitrile and the large variations in signal positions in DMF, only methanol and DMSO seem to be suitable as solvents for the urine matrix—besides water, of course.

The replicates used were only technical replicates of a single batch of urine. To simulate differences in pH and salt concentration between individuals to some extent, new urine samples of the same batch were prepared and various acid, base, and salt solutions were added in a controlled way.

**Figure 4:**
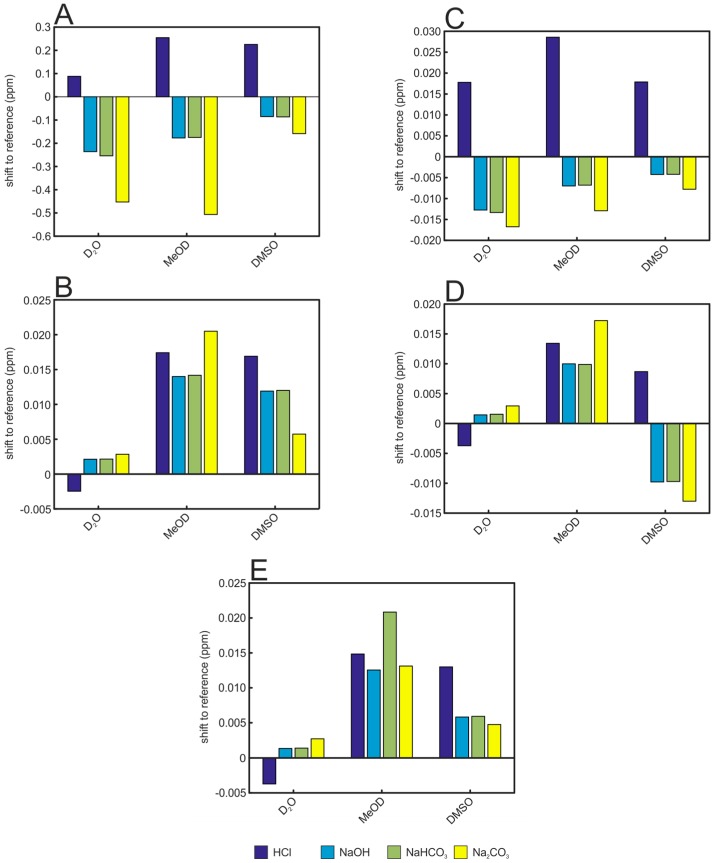
Effect of various acid, base, and salt solutions on the signal positions of selected metabolites in urine samples. Shifts are shown relative to the samples without any acid/base/salt solution added. (**A**) histidine; (**B**) hippurate; (**C)**: creatinine; (**D)** alanine; (**E**) lactate.

A first effect concerning all added solutions tested is directly visible in the positions of the signals (Figure 4). All signals are shifted, but the strength and direction of the shift depends on the solvent and the added acid/base/salt. In water, the addition of HCl shifts the selected signals in the opposite direction compared to NaOH and the basic salts NaHCO_3_ and Na_2_CO_3_. Furthermore, the direction of the shifts for one solution is not always the same for the five selected metabolites. While the addition of HCl to the aqueous samples shifts the signals of histidine and creatinine to higher ppm values, it shifts the signals of lactate, alanine, and hippurate to lower ppm values. For hippurate and lactate in MeOD and DMSO matrices instead, the signal positions are always shifted to higher ppm values for all added solutions. Again, the effect is strongest for the very pH-sensitive metabolite histidine which can be observed within an area of 0.54 ppm for water, 0.76 ppm for MeOD, and 0.38 ppm for DMSO (Figure 4).

**Figure 5:**
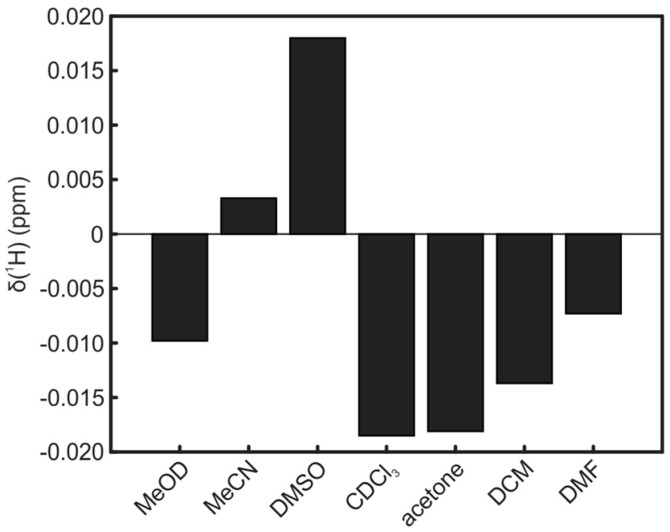
Chemical shift of TMS relative to TSP for all organic solvents.

The reported shifts for all solvents are relative to TSP. Since the standard for organic solvents is usually TMS, reference experiments were performed to get also the shift of TMS relative to TSP. For the used solvents, shifts in positive and negative directions up to 0.02 ppm could be observed (Figure 5).

The solvents’ ability to dissolve the urine matrix correlates directly with the empiric polarity determined by solvent shifts in absorption spectra (Table 3) [23]. The only exception that does not fit into this row is acetonitrile. Apparently, next to polarity, the ability to form hydrogen bonds is important as well.

The deviations in signal positions are minimal for water both when exchanging the solvent and when adding an acid, base, or salt solution. Only when adding the acid/base/salt solutions to DMSO, the shift area is smaller for creatinine and histidine. As addition of these solutions also can be seen as one way to simulate chemical shift changes among different urines, it can be concluded that the salt dependence of unbuffered (!) water is always better compared to other solvents. Using buffer, the situation for water should even improve significantly.

In essence, the current study presents another argument for investigating urine in its original aqueous matrix. While it comes with the least processing of samples, it has the least solubility problems, and has a multitude of chemical shift data available in corresponding databases, the minimum variation in chemical shift positions as compared to other solvents are all clear indications for H_2_O/D_2_O as the preferred NMR solvent of urine samples.

## 3. Materials and Methods

Spot urine of a single healthy male human was collected in the morning and directly used for analysis. To test the reproducibility, five samples for each solvent were prepared individually. In a 15 mL centrifuge tube, 1000 μL urine and 111 μL TSP (3-(trimethylsilyl)-2,2,3,3-tetradeuteropropionic acid) solution (0.1 wt % in D_2_O) were mixed, frozen in liquid nitrogen, and lyophilized for two days. The residue was mixed with 1000 μL of a deuterated solvent (D_2_O, methanol-*d*_4_ (MeOD), acetonitrile-*d*_3_ (MeCN), dimethyl sulfoxide-*d*_6_ (DMSO), CDCl_3_, acetone-*d*_6_, dichloromethane-*d*_2_ (DCM), dimethyl formamide-*d*_7_ (DMF)) and shaken for 10 min (*neolab* Intelli Mixer, program: u1, speed: 85 rpm). The mixture was transferred into a 2 mL microcentrifuge tube and centrifuged for 10 min at 14,000 rpm. 600 μL of the supernatant were transferred into a 5 mm standard NMR tube.

To test the stability of signal positions, also samples with added acid, base, or salts were compared, for which three samples for each acid/base/salt were prepared individually. In a 15 mL centrifuge tube, 810 μL urine and 90 μL TSP solution (0.1 wt % in D_2_O) were mixed, frozen in liquid nitrogen and lyophilized for two days. The residue was mixed with 810 μL of D_2_O, methanol-*d*_4_ or dimethyl sulfoxide-*d*_6_ and 81 μL of an acid/base/salt solution (0.1 M HCl (aq.), 0.1 M NaOH (aq.), 0.1 M NaHCO_3_ (aq.), 0.1 M Na_2_CO_3_ (aq.)) and shaken and centrifuged as described above. 600 μL of the supernatant were transferred into a 5 mm standard NMR tube.

Standard NMR tubes were purchased from Duran (DURAN Group GmbH, Wertheim, Germany).

All NMR spectra were acquired on a Bruker Avance II 600 MHz spectrometer equipped with a ^1^H-BBI double resonance probe head (Bruker BioSpin GmbH, Rheinstetten, Germany). 1D NOESY experiments with presaturation for water suppression were recorded. A prescan delay of 4 s was used together with a mixing time of 10 ms. Pulse lengths were determined automatically by the Bruker AU program *pulsecal*. 64 k complex data points corresponding to a sweep width of 12,345.6 Hz were recorded. All spectra were treated identically using an exponential apodization function, introducing an additional linewidth of 0.3 Hz. Automated phasing, baseline correction, and referencing was done using the Bruker macro *apk0.noe*.

Integration of spectra was done with AMIX 3.9.10 (Bruker BioSpin GmbH) using the multi integrate tool by summing up all points in specified chemical shift regions. Regions for each metabolite were determined individually for each solvent. The baseline was subtracted individually for each peak by subtracting a linear interpolation background defined by the baseline levels left and right of the signal. Peak positions were determined with TopSpin 3.2 (Bruker BioSpin GmbH).

Peak assignment for all solvents was performed by adding pure substance to the samples and recording spectra before and after the addition.

## 4. Conclusions

In this article, we describe a systematic study testing the hypothesis if organic solvents might lead to a reduced variability of chemical shift positions of selected compounds. Unfortunately, the clear answer is that water seems to have the best NMR reproducibility of chemical shifts as compared to organic solvents. For nonpolar solvents, generally only a subset of substances is detected. MeOD, DMSO, and DMF as polar organic solvents are in principle applicable with urine in terms of solubility of metabolites. Variations in chemical shifts and signal integrals, however, are significantly higher than in D_2_O. The same result is found when various acid, base, or salt solutions are added to the samples. We therefore have to conclude that the exchange to organic solvents for urine samples is not advantageous in terms of chemical shift stability and general spectral variations.

## Figures and Tables

**Table 1:** Relative standard deviation of selected integral values.

Solvent	Hippurate	Creatinine	Lactate	Histidine	Alanine
D_2_O	0.86%	1.15%	1.20%	1.70%	1.35%
MeOD	2.76%	3.29%	0.60%	7.01%	1.99%
DMSO	6.92%	11.16%	12.01%	7.30%	6.64%
DMF	1.10%	1.60%	7.03%	74.12%	9.37%
MeCN	31.83%	27.97%	43.27%	-	-
Acetone	26.84%	4.66%	10.74%	-	-
CDCl_3_	15.65%	-	-	-	-
DCM	4.83%	-	-	-	-

**Table 2:** Relative integrals to water as solvent.

Solvent	Hippurate	Creatinine	Lactate	Histidine	Alanine
MeOD	1.30	1.30	0.96	1.64	1.01
DMSO	1.18	1.10	0.46	1.39	0.65
DMF	1.34	1.59	0.38	0.20	0.59
MeCN	0.22	0.15	0.10	-	-
Acetone	0.32	0.11	0.09	-	-
CDCl_3_	0.13	-	-	-	-
DCM	0.08	-	-	-	-

**Table 3:** Relative polarity of the used solvents [23].

	H_2_O	MeOD	MeCN	DMSO	DMF	Acetone	DCM	CHCl_3_
*E*_T_^N^	1.000	0.762	0.460	0.444	0.386	0.355	0.309	0.259

## Supplementary Materials

Supplementary materials can be found at www.mdpi.com/2218-1989/6/3/27/s1. Table S1: Mean value and standard deviation of the creatinine signal at 3 ppm, Table S2: Mean value and standard deviation of the hippurate signal at 7.5 ppm, Table S3: Mean value and standard deviation of the lactate signal at 1.3 ppm, Table S4: Mean value and standard deviation of the histidine signal around 8.2 ppm, Table S5: Mean value and standard deviation of the alanine signal at 1.5 ppm. Figure S1: T_1_ relaxation times of selected metabolites and solvents.
