# Supplementary material for: NMR Chemical Shift Ranges of Urine Metabolites in Various Organic Solvents
Source: Metabolites. 2016 Sep 2;6(3):27. doi: 10.3390/metabo6030027 (PMC5041126; doi:10.3390/metabo6030027)
Supplement: Supplementary file 1 [file metabolites-06-00027-s001.pdf]

Supplementary Materials: NMR Chemical Shift Ranges of Urine Metabolites in Various Organic Solvents

Benjamin Görling, Stefan Bräse and Burkhard Luy

Tabular Representation of Integrals

Integral values were calculated for the five selected metabolites for all five spectra per solvent by summing up all points in the region. Regions were defined individually for every solvent. Mean values and standard deviations are given in Table S1–S5.

Table S1. Mean value and standard deviation of the creatinine signal at 3 ppm.

Solvent	Mean	Standard Deviation	Deviation in %
**Water**	2.49×10^{8}	2.85×10^{6}	1.15%
**Methanol**	3.23×10^{8}	1.06×10^{7}	3.29%
**Acetonitrile**	3.79×10^{7}	4.24×10^{6}	11.16%
**DMSO**	2.73×10^{8}	4.37×10^{6}	1.60%
**Acetone**	2.83×10^{7}	7.91×10^{6}	27.97%
**DMF**	3.96×10^{8}	1.85×10^{7}	4.66%

Table S2. Mean value and standard deviation of the hippurate signal at 7.5 ppm.

Solvent	Mean	Standard Deviation	Deviation in %
**Water**	3.70×10^{7}	3.17×10^{5}	0.86%
**Methanol**	4.82×10^{7}	1.33×10^{6}	2.76%
**Acetonitrile**	8.15×10^{6}	5.64×10^{5}	6.92%
**DMSO**	4.36×10^{7}	4.81×10^{5}	1.10%
**Chloroform**	4.90×10^{6}	1.56×10^{6}	31.83%
**Acetone**	1.17×10^{7}	3.14×10^{6}	26.84%
**Dichloromethane**	2.82×10^{6}	4.42×10^{5}	15.65%
**DMF**	4.96×10^{7}	2.40×10^{6}	4.83%

Table S3. Mean value and standard deviation of the lactate signal at 1.3 ppm.

Solvent	Mean	Standard Deviation	Deviation in %
**Water**	4.31×10^{6}	5.18×10^{4}	1.20%
**Methanol**	4.15×10^{6}	2.51×10^{4}	0.60%
**Acetonitrile**	4.36×10^{5}	5.24×10^{4}	12.01%
**DMSO**	1.96×10^{6}	1.38×10^{5}	7.03%
**Acetone**	3.89×10^{5}	1.68×10^{5}	43.27%
**DMF**	1.66×10^{6}	1.78×10^{5}	10.74%

Table S4. Mean value and standard deviation of the histidine signal around 8.2 ppm.

Solvent	Mean	Standard Deviation	Deviation in %
**Water**	3.80×10^{6}	6.44×10^{4}	1.70%
**Methanol**	6.24×10^{6}	4.37×10^{5}	7.01%
**DMSO**	5.27×10^{6}	3.85×10^{5}	7.30%
**DMF**	7.43×10^{5}	5.51×10^{5}	74.12%

Table S5. Mean value and standard deviation of the alanine signal at 1.5 ppm.

Solvent	Mean	Standard Deviation	Deviation in %
**Water**	7.42×10^6	1.00×10^5	1.35%
**Methanol**	7.50×10^6	1.49×10^5	1.99%
**DMSO**	4.82×10^6	3.20×10^5	6.64%
**DMF**	4.35×10^6	4.08×10^5	9.37%

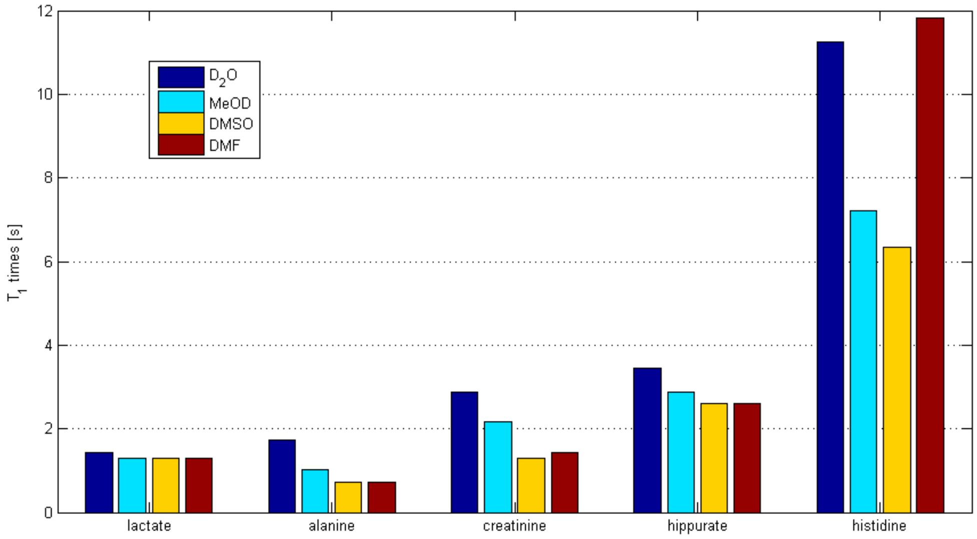

Figure S1. T_1 relaxation times of selected metabolites and solvents. The T_1 times in D_2O are larger than 2 s for most of the metabolites. In MeOD, DMSO, and DMF relaxations times are significantly shorter with the exception of histidine in DMF.
